# A History of Vena Cava Interruption to Treat Pulmonary Embolism: The Ben Hogan Case

**DOI:** 10.31486/toj.26.0038

**Published:** 2026

**Authors:** Theodore N. Pappas, Tiffany Lim, Justin Barr

**Affiliations:** ^1^ Department of Surgery, Duke University, Durham, NC; ^2^ The University of Queensland Medical School, Ochsner Clinical School, New Orleans, LA; ^3^ Ochsner Transplant Institute, Ochsner Clinic Foundation, New Orleans, LA

## INTRODUCTION

Ben Hogan (1912-1997) was one of the most successful professional golfers of the mid-20th century. On February 2, 1949, while driving home from a golf tournament in Phoenix, Arizona, he was involved in a head-on collision with a bus. Hogan sustained multiple orthopedic injuries, including a fractured pelvis and left ankle. During his recovery, he developed deep vein thromboses (DVTs) in his injured leg, complicated by pulmonary emboli. To prevent recurrent pulmonary emboli, Alton Ochsner ligated his infrarenal vena cava on March 3, 1949; Hogan was discharged home a month later. He recovered during the following year and went on to have a Hall of Fame golf career, winning a total of 69 Professional Golfers’ Association (PGA) tournaments, including 9 major tournaments, before he retired in 1971. In an article about the importance and value of inferior vena cava ligation for pulmonary embolism prevention, Ochsner referred to his famous operation on Hogan, quipping, “A number of my friends have threatened to have their cavae ligated prophylactically, in order to improve their golf game.”^1^ We review Hogan's clinical course and discuss the history of vena cava interruption for the management of DVT.

## BEN HOGAN: EARLY LIFE

Hogan was born on August 13, 1912, in Stephenville, Texas, to parents who owned a blacksmith shop in nearby Dublin, Texas. As automobiles became commonplace, the blacksmith trade diminished, Hogan's father's business failed, and the family went into debt. The family sold their home and moved to Fort Worth, Texas, where Hogan's father shot himself with his young son in the next room. Hogan worked from an early age to help support the family. At age 11, he took a job as a caddie at Glen Garden, a country club in Fort Worth. He developed an interest in golf and played during his free time, demonstrating the necessary diligence to improve his game. He won his first tournament, the Cleburne Invitational at the Cleburne Country Club, when he was in high school. In 1929, Hogan dropped out of high school to turn professional.^2-5^

Hogan met his wife Valerie when they were 12 years old, and they married in 1935.^6^ His wife actively supported his golfing career, sometimes serving as his assistant, manager, and secretary. Hogan initially struggled with his professional game until 1938 when he had several top-10 finishes on the PGA tour, including 9th place in the 1939 Masters Tournament.^3^ By the end of the 1939 golf season, he had 16 top-10 finishes and was seventh on the money list for the PGA.^7,8^ His breakout year occurred in 1940 when he won 4 tournaments, won the Vardon Trophy for having the lowest average score for the year, and was the PGA tour money leader.^9^ His tour performance was equally remarkable in 1941 and 1942: he finished 3 seasons in a row as the tour's money leader.^10^ Hogan was drafted into the United States Army Air Forces in 1943 and served until he was honorably discharged in August 1945.^11^ From 1946 until 1949, he continued his dominant performance on the PGA tour: money leader in 1946; winner of 7 tournaments in 1947; and winner of 10 tournaments in 1948, including the PGA and US Open championships.^12^

## MOTOR VEHICLE ACCIDENT

**Figure 1.**
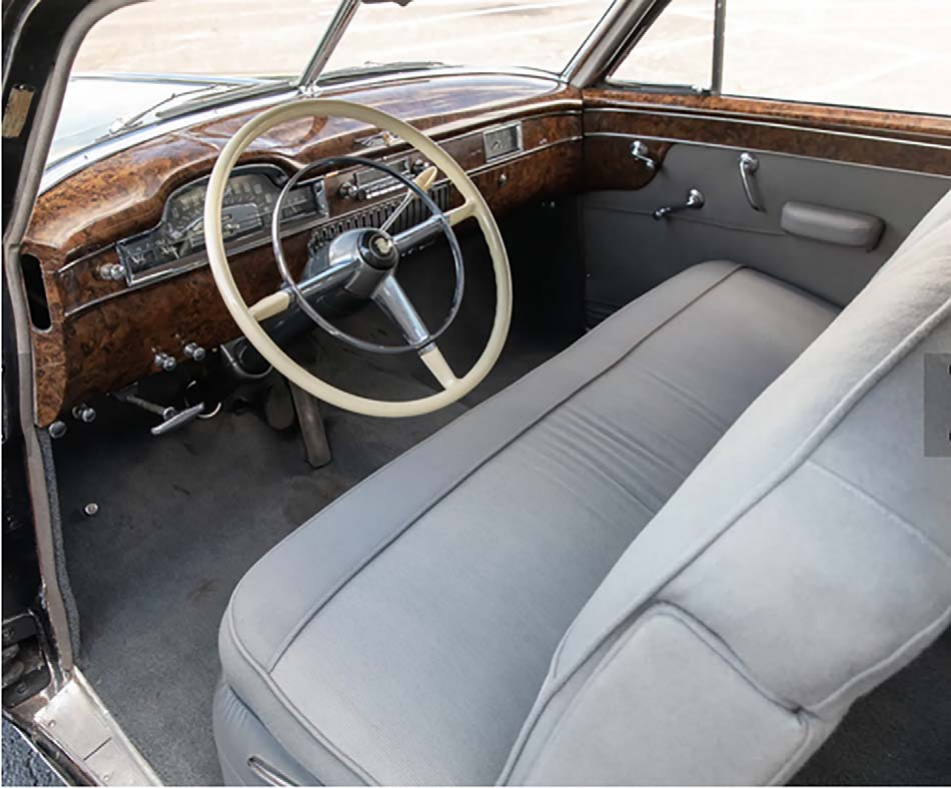
Interior front seat of a 1949 Cadillac Series 62 Sedan similar to the car Ben Hogan was driving when he had a head-on collision with a Greyhound bus. Note the lack of seat belts that were not standard equipment in Cadillac vehicles until 1963. The bench-like front seats were standard until 2003 when bucket seats became the standard in all new Cadillacs. In 1959, Cadillac began offering bucket seats as an upgrade option.

**Figure 2.**
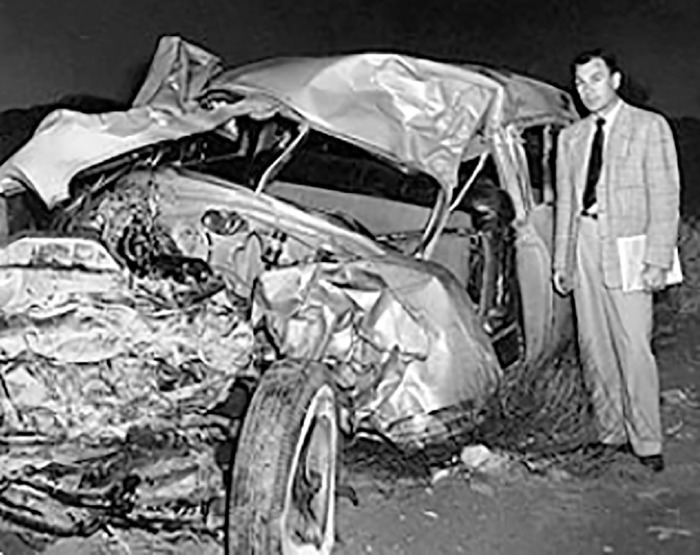
**Wreckage of Ben Hogan's 1949 Cadillac after colliding with a Greyhound bus on February 2, 1949, near Van Horn, Texas.** (Photo from Ben Hogan's Facebook page.)

In January 1949, Hogan won the Bing Crosby Open at Pebble Beach,^13^ won the Long Beach Open,^14^ and finished second in the Phoenix Open.^15^ After the Phoenix tournament, Hogan and his wife began driving back to their home in Fort Worth. Continuing the journey on February 2, Hogan's deceased father's birthday, Hogan and his wife encountered heavy fog, and a Greyhound bus plowed head-on into their new Cadillac at exactly 8:30 am. The bus driver was attempting to pass a truck on a 2-lane bridge and never saw Hogan's vehicle in the fog. As the bus struck the left front of the car, Hogan threw himself to his right, across his wife's lap. In 1949, Cadillacs did not have seat belts, and the front seats were configured as bench (not bucket) seats (Figure 1). The lack of seat belts and the bench seat allowed Hogan to move his body to the passenger side of the seat as the impact occurred. This shift protected his wife from serious injury and saved Hogan's life. The head-on collision pushed the motor and steering column through the driver's side seat (Figure 2); the steering wheel clipped and fractured Hogan's left shoulder.^4,8^

Hogan was knocked unconscious; his pelvis, left ankle, left clavicle, and right rib were fractured; and his left eye and bladder were contused.^16^ Several news reports initially reported his death, and the Associated Press later distributed his obituary to media outlets. Hogan's wife suffered only minor injuries, and the passengers of the bus escaped unscathed. The ambulance required 90 minutes to arrive and another 3 hours to reach the nearest hospital, during which time the golfer fluctuated in and out of consciousness. Hogan was admitted to Hotel Dieu Hospital in El Paso, Texas, where physicians stabilized him and applied casts to his fractures.^4^

During the next 10 days, Hogan steadily improved.^17^ A tremendous outpouring of support from celebrities such as Katharine Hepburn to anonymous fans buoyed his spirits, and as the press trumpeted his heroic lunge to save his wife, Hogan's previously sterile and downright ornery public personality gave way to a more human and (slightly) more vulnerable mien. The near-death experience also prompted him to be baptized.

By mid-February 1949, Hogan was ready for discharge but then sustained a setback on February 18: worsening swelling in his left leg. His doctors diagnosed phlegmasia alba dolens and initiated heparin.^18,19^ On February 27, Hogan developed chest pain and was diagnosed with pulmonary emboli.^20,21^ Hogan's physicians called Dr. Nelson W. Barker of the Mayo Clinic, an expert in vascular disease, clotting, and anticoagulation, who helped confirm the diagnosis of pulmonary emboli.^20^ Barker, a pioneer in the radiologic diagnosis of pulmonary embolism and DVT,^22^ recommended surgical intervention and suggested calling Alton Ochsner, professor of surgery at Tulane University.^20^

Alton Ochsner (1896-1981) was born in South Dakota and graduated from the University of South Dakota in 1918.^23^ He earned his medical degree in 1920 from Washington University School of Medicine in St. Louis, Missouri, before pursuing additional training in surgery, first in Chicago, Illinois, under the guidance of his cousin A. J. Ochsner and then in Europe under Paul Clairmont in Zurich, Switzerland, and Viktor Schmieden in Frankfurt, Germany. Ochsner joined the faculty of the University of Wisconsin in 1926 but a year later accepted the offer to chair the Department of Surgery at Tulane University in New Orleans, Louisiana. He established a busy clinical practice at Charity Hospital in New Orleans, and then, in 1942, founded the Ochsner Clinic with 4 other members of the Tulane faculty.^24^ Ochsner remains best known for his early position in 1939 that tobacco smoking was linked to lung cancer. During his career, Ochsner served as the president of the American Cancer Society and the American College of Surgeons. He developed particular expertise in treating vascular conditions such as phlegmasia cerulea dolens and DVT.^1,25^

Ochsner agreed to travel to Texas and help Hogan. However, because of severe weather, he had difficulty securing a commercial flight to El Paso. Hogan's family contacted US Air Force Brigadier General David William Hutchison^26^ to arrange a flight for Ochsner, and the general sent a B-29 bomber to pick up Ochsner and ferry him to El Paso. Such transport is highly irregular, and Hutchinson's motivation for deploying an Air Force bomber for this mission remains unclear. Some sources claim Hogan and Hutchinson knew each other from the Second World War or were golfing partners after the war; other sources report that Hogan's brother Royal made the connection or that Hogan's wife Valerie knew the general. Regardless, Ochsner arrived on March 3 and promptly took a nap, having been awake all night coordinating travel logistics.

Once refreshed, Ochsner offered to ligate Hogan's vena cava to reduce the risk of future pulmonary emboli. Ochsner was quite clear about the likely side effects of swelling and warned Hogan that he might never play golf again.^4^ Hogan was transfused with 3 units of blood in anticipation of the operation that started just after 6:30 pm, only a few hours following Ochsner's arrival. During the 2-hour laparotomy, Ochsner ligated Hogan's infrarenal vena cava without complication. Some hours later, at 12:30 am on March 4, Ochsner flew back to New Orleans.^27^

Hogan's postoperative course was uncomplicated but prolonged. He was allowed out of bed for the first time on March 14, postoperative day 11,^28^ and was discharged on April 1, 1949.^29^ He traveled by train to his home in Fort Worth, where he continued to improve.^29^ Ochsner saw Hogan for follow-up in New Orleans on April 30 and noted that he had no postoperative complications.

## RETURN TO GOLF AND SUBSEQUENT MEDICAL HISTORY

After intense rehabilitation, Hogan played his first round of golf on December 10, 1949, and entered his first tournament since the crash, the Los Angeles Open, in January 1950, finishing second to Sam Snead. He tied for fourth place in the April Masters Tournament and won the Greenbrier Pro-Am in May 1950. He won the 1950 US Open held at Merion Golf Club in a 3-way playoff. Hogan continued to win tournaments throughout the early 1950s, including the 1951 Masters, 1951 US Open, 1953 Masters, 1953 US Open, and 1953 British Open Championship in Scotland. Hogan won his final tournament in 1959 and played in his last PGA event in 1971, at age 58.^3^

Hogan was a chronic smoker (2 packs per day) until his 80s. He ultimately underwent 4 operations on his left shoulder, presumably related to sequelae of the car accident. An appendix rupture in 1987 required an appendectomy that was complicated by pneumonia. His colon was removed in 1995 for cancer. Hogan developed Alzheimer disease in his final years and died from pneumonia following a stroke in 1997 at age 84.^3,4,8^

## PREVENTION OF PULMONARY EMBOLISM THROUGH CAVAL INTERRUPTION

Surgeons had long recognized the fatal potential of postoperative thrombosis, particularly following orthopedic surgery.^30^ In the early 20th century, preventive measures included elevating the lower extremities, muscle exercises, and early mobility. Some authors recommended drugs such as calcium, thyroid hormone, or leech application, but these treatments were uncommon and ineffective.^31^ Heparin had recently become commercially available and accepted for the treatment of blood clots but was rarely used for prevention.^32,33^ In 1908, Friedrich Trendelenburg first attempted his eponymous operation to extract a pulmonary embolus surgically, but the patient died, as did the next 20 patients on whom others operated.^34^ Trendelenburg's student, Martin Kirschner, first successfully performed the surgery in 1924.^34^ The rare instance of patient survival—surgeon Edward Churchill famously wrote in 1934, “the procedure could perhaps be more properly termed an immediate post-mortem examination than a surgical operation”^35^—underscored the importance of prophylaxis.

In the 1940s, the standard method for preventing DVTs from embolizing to the pulmonary arteries involved interrupting the vena cava. Boston vascular surgeon John Homans first proposed “venous ligation above the thrombus” in patients with DVT, ligating a femoral vein in 1934.^36^ The practice spread. A 1955 report of 369 patients undergoing bilateral femoral division between 1943 and 1955 at Boston City Hospital demonstrated a 2.1% mortality for those undergoing the operation compared to 37% mortality for those treated conservatively.^37^ Electively ligating the vena cava for proximal iliac clots represented the next logical step, which Collins et al classically described in 1943.^38^ Homans supported this technique for clots above Poupart's ligament, particularly in bilateral disease, helping precipitate a change in practice with his 1944 ligation.^39^ By the late 1940s, inferior vena cava ligation for pulmonary embolism prevention in the setting of DVT was becoming the standard of care.^40,41^

Alton Ochsner developed particular expertise with venous clotting disorders and authored several papers on the topic with his young protégé, Michael DeBakey.^42^ Ochsner pointedly distinguished phlebothrombosis from thrombophlebitis, with therapeutic implications.^43^ He argued that phlebothrombosis represented unprovoked venous clot that often required operative intervention because the clot floated freely and frequently led to fatal embolism. For these patients, Ochsner recommended preemptive venous ligation. In contrast, patients such as Ben Hogan who developed thrombosis following trauma or other injury to the venous system had adherent clot that did not inherently require prophylactic venous ligation. However, when nonfatal pulmonary emboli occurred in patients with thrombophlebitis, Ochsner and DeBakey recommended urgent thrombectomy with venous ligation to prevent a fatal recurrent embolus.^1,44,45^ Like many of his contemporaries. Ochsner recommended surgical intervention based on history, physical examination, and chest x-ray findings alone and did not require contrast phlebography.^44^

Despite the routine use of heparin, Ochsner aggressively recommended venous ligation above the clot in all cases “[b]ecause the administration of anticoagulants will not prevent the detachment of clots.”^44^ He wrote, “Because the thrombus in phlebothrombosis is only loosely attached to the vein wall and can be detached by any slight exertion, it is my belief that immediate surgical intervention should be undertaken as soon as the diagnosis of phlebothrombosis is made.”^44^ Ochsner and DeBakey recognized the severity of caval interruption but as early as 1943 deemed it “lifesaving.”^46^ Ochsner's documented aggressive approach to this disease explains why, when contacted about Hogan's clinical condition, he traveled to El Paso as soon as he could get a flight and operated within hours of arrival.

By the 1960s, vena cava ligation had become an established method to prevent lower extremity thrombosis from sending clots to the lungs but was associated with major morbidity. In 1955, Ralph F. Bowers and Samuel M. Leb clearly documented long-term complications resulting from interrupted venous return to the heart, findings soon echoed by other authors.^47-49^ Following his surgery, Hogan suffered chronic edema of his lower extremities; he routinely wore compression wraps on both legs when playing golf and struggled when walking long distances. These complications led to a variety of technical modifications intended to preserve some blood flow while blocking the clot. Moretz et al proposed a lumen-narrowing clamp^50^; Spencer et al suggested suture plication^51^; DeWeese and Adams^52,53^ and Miles et al^54^ introduced serrated clips. Ultimately, a dozen technical variations appeared in the literature.^55^

These modifications, however, frequently led to similar complications.^56^ No randomized clinical trial compared caval ligation vs the theoretically less-invasive methods, and retrospective studies presented conflicting results regarding efficacy and complications of plication vs ligation vs clip, the choice of which remained a subject of debate in the surgical literature.^56-59^ Alternatives remained limited. By 1960, thrombolytic therapy had emerged as a possible treatment for pulmonary embolism but had little role in prevention.^60^ Lazar Greenfield had success with an intravenous approach that limited operative morbidity, but because the cava was still closed, the Greenfield procedure also caused long-term sequelae.^61^ Despite advances in medicine, venous ligation remained the standard of care for more than 4 decades. In 1974, former President Richard Nixon developed a DVT that prevented him from testifying in the Watergate trial; his surgeons placed a Miles clip across his left iliac vein and received some criticism for not ligating the inferior vena cava entirely.^62^ Caval interruption, in one form or another, continued into the 1980s.

Intraluminal inferior vena cava filters eventually replaced ligation, clips, and plication. First described by Mobin-Uddin et al in 1969,^63^ filters replaced ligation as the standard of care largely because of the work and advocacy of Lazar Greenfield. Greenfield et al created a transvenous filter that precluded the need for anesthesia or celiotomy while maintaining flow through the cava,^64^ and studies soon demonstrated the superiority of the transvenous filter compared to the Mobin-Uddin et al device.^65^ Variations quickly appeared, with more than 20 US Food and Drug Administration–approved models available for implantation.^66,67^ Because filters seemed to minimize complications while effectively preventing blood clots, filter use increased rapidly.^68^ In 1979, approximately 2,000 patients in the United States underwent filter placement; by 1999, the number had climbed 25-fold to 49,000.^69^

In 1998, approximately 30 years following filter introduction, Decousus et al performed the first randomized clinical trial comparing inferior vena cava filters against systemic anticoagulation.^70^ Decousus et al found that in the short-term, filters markedly reduced the risk of pulmonary embolism compared to heparin, but that over a 2-year follow-up, mortality was the same in both groups. Critically, the rate of recurrent DVTs in the filter group (20.8%) was almost double the rate of DVTs in the heparin alone group (11.6%),^70^ highlighting one of the main adverse effects of filters and helping to clarify specific indications for their use, notably in patients unable to tolerate therapeutic anticoagulation.

Thromboses and other complications such as migration, perforation, vascular injury, and hemorrhage^71,72^ catalyzed the development of retrievable filters in the early 2000s.^73-75^ However, retrieval is potentially fraught with danger, including perforation of the inferior vena cava and/or surrounding structures, fracture and subsequent embolization of the filter, stenosis at the prior filter site, and complications related to internal jugular vascular access.^76^

## CONCLUSION

As a result of a horrific automobile accident, Ben Hogan suffered serious injuries complicated by life-threatening DVTs. Alton Ochsner performed the standard operation for the era. Although Hogan tolerated the procedure and went on to a highly successful golf career, he suffered the rest of his life from swollen, painful legs, a known, if sometimes minimized, complication of inferior vena cava ligation. Hogan did not, however, die from a pulmonary embolism. Decades later, superior strategies involving retrievable filters and systemic anticoagulation—first with heparin, later with coumadin, and then with direct oral anticoagulants—supplanted vessel ligations. These modern therapies also have drawbacks, and surgical patients in particular continue to die from pulmonary emboli. Identifying the perfect balance between effective prophylaxis and no adverse effects remains a never-ending quest, much like a perfect round of golf.
